# Combined treatment with bacteriophages and sodium hypochlorite against *E. faecalis* biofilm in an *in vitro* root canal model with evaluation of bacterial resistance

**DOI:** 10.1080/20002297.2026.2687225

**Published:** 2026-06-14

**Authors:** Konstantin Steffen, Kerstin Bitter, Sebastian Schlafer, Sebastian Paris, Marie Bauer

**Affiliations:** a Charité – Universitätsmedizin Berlin, Department of Operative, Preventative and Paediatric Dentistry, Berlin, Germany; b Department of Operative Dentistry and Periodontolgy, Martin-Luther University Halle-Wittenberg, Halle, Germany; c Aarhus University, Department of Dentistry and Oral Health, Section for Oral Ecology, Cariology, Aarhus, Denmark

**Keywords:** E. faecalis, bacteriophage, bacterial resistance, biofilm, root canal treatment

## Abstract

**Aim:**

To assess the time-dependent antimicrobial activity of a newly isolated bacteriophage against *Enterococcus faecalis* biofilms in two *in vitro* models, aloneand combinedwith sodium hypochlorite, followed by the evaluation of potential resistance development.

**Methodology:**

Bacteriophage CUB_EF02 was isolated from sewage water using *E. faecalis* ATCC 29212 as host, visualized by TEM, and sequenced. Its antimicrobial effect against biofilms grown on porous glass beads was assessed in a time-killing assay. Additionally, 10-day-old biofilms were established in 36 extracted single-rooted human teeth and treated with 0.9% NaCl, 1% NaOCl, phages, or their combination. Dentine-adherent biofilm was quantified by plate counts at baseline, after treatment, and after 48 h re-incubation. Logarithmic reduction factors were analyzed using one-way ANOVA and Tukey HSD. Phage resistance was evaluated by Top-Agar overlay spot assay.

**Results:**

Phage treatment reduced biofilm by 1.35 ± 0.2 SD-log after 6 h compared to control. Irrigation of the root canals using NaOCl achieved a significantly higher reduction (LRF_red._ = 2.98 ± 0.77 SD) than phages alone(LRF_red._ = 1.50 ± 0.23 SD). Combined treatment showed the strongest antimicrobial effect (LRF_red._ = 4.05 ± 1.17 SD). Bacterial regrowth occurred in all groups. Resistance developed after phage incubationfor ≥6 h.

**Conclusion:**

Bacteriophages represent a promising adjunctive in root canal disinfection, but remain less effective than NaOCl and may inducebacterial resistance.

## Introduction

Secondary infections after endodontic treatment occur with a prevalence of up to 15% of teeth with pre-operative apical periodontitis, even if the treatment has followed proper standards [1–3]. This often leads to surgical intervention such as apicoectomy or tooth extraction [4]. Possible reasons include renewed bacterial colonisation of the root canal system due to leaking post-endodontic restorations, secondary caries, or endodontic periodontic lesions [5]. Re-inflammation can also occur if microorganisms remain in the canal system [6]. This may be the case if the canals have been insufficiently disinfected [7].

A revision treatment is usually accompanied by an increased preparation size to ensure sufficient removal of the remaining biofilm [8]. However, increased mechanical preparation can lead to perforations and severe weakening of the root, which in turn can promote root fractures [9]. Chemo-mechanical preparation also has its weaknesses in terms of biofilm reduction. Due to the complexity of the root canal system, microorganisms can remain in lateral canals, ramifications, and isthmuses [10–12]. The anatomy of the tooth root also plays an important role. In oval canals, for example, up to 20-40% of the canal wall remains unprepared [13,14].

According to current knowledge, various types of bacteria are involved in the development of pulpitis, pulp necrosis, and apical periodontitis [15]. The most frequently detected pathogens contributing to the development of primary apical periodontitis include *Fusobacterium*, *Pseudoramibacter*, *Novosphingobium*, *Ralstonia*, and *Bacteroides* [16]. Manoil et al., 2020 showed that *E. faecalis*, a Gram-positive, facultative anaerobic bacterium that naturally exists in the intestinal flora of mammals, including humans [17], is an opportunistic pathogen of primary apical periodontitis, colonising the pulp chamber via caries cavities, but is not directly involved in the development of apical periodontitis [18,19]. *E. faecalis* is, however, regarded as an important factor in the development of secondary periapical infections [20], as it is nine times more likely to be found in secondary than in primary lesions [21] and shows prevalences of up to 77% in post-treatment root canal infections [22,23]. Clinical studies have shown that *E. faecalis* survives the primary root canal treatment by hiding in dentinal tubules, lateral canals, or apical ramifications. Due to its resistance to high pH and common endodontic irrigants such as EDTA or sodium hypochlorite, *E. faecalis* is difficult to remove from the root canal. It forms biofilms that are firmly attached to the root dentine and in which the cells are encased in a protective extracellular matrix that shields the bacteria from the inflammatory defence system of the human body, from disinfection irrigants and from antibiotics [24]. *E. faecalis* is thus found in about half of the non-healing periapical lesions with proven resistance against antibiotics [25]. Especially through the formation of biofilm, *E. faecalis* is described as a hard-to-eradicate pathogen in dentistry [21]. The bacteria attach themselves to the dentin and produce an extracellular matrix containing polysaccharides, proteins, and DNA [24,26]. This biopolymeric matrix protects the bacteria from antibiotics, disinfection irritants, phagocytosis, and other components of the inflammatory defence system of the human body [24]. Due to the complex anatomy of teeth and the formation of intrinsically resistant biofilms, complete disinfection of the root canal system cannot always be achieved with the current gold standard of chemo-mechanical root canal preparation (mechanical instrumentation combined with ultrasonically activated irrigation using NaOCl and EDTA). Apical parts of the canal system and lateral canals, in particular, may be insufficiently disinfected and hence be the source of continued infection [27]. These shortcomings call for alternative therapeutic strategies to support conventional disinfection protocols.

The use of bacteriophages (also referred to as phages) against bacteria was previously described [21,28] and could be an alternative therapeutic approach to target *E. faecalis* biofilm. Phages are viruses that attach selectively to specific molecules on the surface of bacteria. They inject their genetic material and replicate within their host [28,29]. After infection and replication, they force the host to lyse and spread their progeny [30]. Through this specific cycle, bacteriophage treatment of bacterial infections can lead to the destruction of biofilms [29]. Particularly important is the fact that phages possess countermeasures to overcome the biofilm barrier, by utilising biofilm matrix-degrading enzymes [31].

Efficient and mandatory lytic phages appear to be promising candidates for therapy against Gram-positive pathogens [32]. For this reason, numerous bacteriophages have already been studied as therapeutic agents to combat bacterial infections, for example against *Staphylococcus aureus* [33,34], *Listeria monocytogenes* [35], and *E. faecalis* [36]. In addition, Khalifa et al. 2016 [21] and Shukla et al. 2025 [37] demonstrated that bacteriophages can significantly reduce monospecies *E. faecalis* biofilms in an *ex vivo* root canal model. However, Basak Erol et al [38]. showed that NaOCl inhibits the activity of bacteriophages when applied simultaneously, and that biofilm regrowth may occur after an initial reduction.

To explore this issue further, we tested the antimicrobial effect of a phage against E. faecalis biofilms in two distinct models *in vitro* and experimentally investigated the time-dependent antibiofilm effect with subsequent evaluation of phage resistance to simulate a possible endodontic treatment. This study sought to determine the optimal incubation time of the newly isolated bacteriophages to achieve maximal bacterial reduction. We aimed to assess the antimicrobial effect, expressed as the logarithmic reduction factor, of sodium hypochlorite, both individually and in combination with isolated bacteriophages, against *E. faecalis* biofilms established in the root canal of human teeth *in vitro*. The null hypothesis states that there is no significant difference between the irrigation protocols applied and the measured logarithmic reduction factor. In addition, the bacteria of the host strain were examined for possible development of phage resistance after phage incubation.

## Materials and methods

The manuscript of this laboratory study has been written according to the Preferred Reporting Items for Laboratory studies in Endodontology (PRILE) 2021 guidelines [39]. Apart from the fluorescence microscopy analysis, performed by (SS), the laboratory work was performed by one operator (KS) under sterile conditions to standardise the experimental conditions.

### Bacterial strains

The ATCC 29212 *E. faecalis* host strain (for simplification, it was designated EF02) was used in this study. Bacteria were stored in a 25% glycerol solution at −80 °C in a low-temperature freezer (V.I.P.™ Series −86 °C Ultra Low-Temperature Freezer, SANYO, Moriguchi, Japan). From these glycerol stocks, liquid overnight cultures (ONC) were established in Tryptic Soy Broth (TSB) (22092-500G, Merck KGaA, Darmstadt, Germany). All cultures were incubated at 37 °C under atmospheric conditions, unless otherwise stated.

### Bacteriophage isolation

To detect lytic bacteriophages against EF02, various effluent samples from the Ruhleben sewage treatment plant in Berlin were screened. Fifty µL of the ONC from the host strains (10^8^ CFU per ml) were added to each of the sewage samples and incubated for 24 h (T6420, Thermo Fisher Scientific Inc., Massachusetts, USA) on a rotary shaker at 150 rpm (KS 260 basic Schüttler, IKA GmbH & CO. KG, Staufen, Germany). Subsequently, the samples were centrifuged (Centrifuge 5430 R, Eppendorf SE, Hamburg, Germany) for 20 min at 6512 × g and filter-sterilised using a 0.22 μm filter (Millex-GP filter unit pore size 0.22 μm, diam. 33 mm, Merck KGaA, Darmstadt, Germany). Afterwards, a molten top agar (MTA) TSB plate was prepared using the double agar technique to create a uniform bacterial lawn. The MTA-TSB (0,6% w/v) consists of 37 g/L TSB and 6 g/L agar (05040-250G, Merck KGaA, Darmstadt, Germany). To produce the MTA-TSB with a bacterial lawn, 100 µL of the ONC from the host strain was added to 5 mL of MTA-TSB in a sterile 15 mL Falcon Tube (352097, Corning, NY, USA). After briefly mixing (Vortex Genie 2TM, Bender & Hobein AG, Zurich, Switzerland), the suspension was poured onto a tryptic soy agar (TSA) plate (22091-500G, Merck KGaA, Darmstadt, Germany). Phage suspension was then spotted onto these plates and incubated for 24 h. After 24 h, the plates were scanned for the presence of lytic zones. For phage isolation, a single plaque was picked using a sterile transfer pipette (Transferpipette 3.5 mL, SARSTEDT, Nürnbrecht, Germany) and subjected to serial plaque purification. Shortly thereafter, a sterile inoculating loop (L200 Ino-Loop™, SIimport, Belœil, Canada*)* was used to streaked over the punctures to spread the bacteriophages across the agar plate. Then, the plates were incubated for 24 h. This step was repeated up to five times until homogenous, clear lytic zones appeared. Next, a sterile pipette (606180, Greiner Bio-One International GmbH, Kremsmünster, Austria) was used to add 5 mL of PBS (GIBCO™ PBS pH 7.4, Thermo Fisher Scientific, Hennigsdorf, Germany) to the agar plates, which were sealed with laboratory film (PARAFILM® M Sealing Film, Bemis, Neenah, USA) and stored at 4 °C (FKv 4310, Liebherr-International Deutschland GmbH, Biberach an der Riß, Germany) for 24 h. Then, the PBS buffer containing the diffused bacteriophages and the MTA-TSB were collected. The suspension was centrifuged for 20 min at 6512 × g and 4 °C, then filter-sterilised using a 0.22 µm filter and stored at 4 °C. Isolated phage was named ‘CUB’ (Charité—Universitätsmedizin Berlin) followed by a number corresponding to the *E. faecalis* host strain. Phages were subjected to plaque picking and serial plaque purification, followed by filtration and centrifugation to minimise bacterial debris and ensure high lytic purity. Chloroform treatment was omitted to preserve phage structural integrity, including tail fibres, and to maintain potential enzymatic activity. Negative controls consisted of a host lawn without phage and a sewage sample processed in parallel without host enrichment. This ensured that plaque formation was attributable to phage activity and not to contamination or spontaneous lysis.

### Bacteriophage production

As previously described [40] a solid-phase propagation was selected over liquid culture amplification to achieve a high phage-titre. The double agar technique was used for phage production. Briefly, TSA plates with a homogeneous EF02 bacterial lawn were created. Two hundred µL of the newly isolated phage stock was spotted on the plates. The phages were spread over the agar plate using a sterile inoculation loop and incubated for 24 h. To each agar plate, 5 mL of PBS was added and subsequently sealed with laboratory film. The plates were incubated for 24 h at 4 °C and 1013 hPa. 24 h later, both the PBS and the top agar were collected from the plates and added to a sterile 50 mL laboratory tube (Centrifuge Tube Ct 50 mL, VWR International GmbH, Dresden, Germany). The suspensions were centrifuged (20 min, 6512 × g, 4 °C) and filter-sterilised using a 0.22 µm filter. Additionally, to detect possible halo formation of the isolated phage, as previously described [41], 10 µL of the phage suspension with a titre of 10^8^ plaque forming units (PFU)/mL was pipetted onto a TSA plate covered with a homogeneous bacterial lawn of EF02 and incubated for 24 h. Halo formation was assessed exclusively in a qualitative manner.

### Bacteriophage titre determination

An eight-fold serial dilution of the CUB_EF02 suspension was performed using 2 mL tubes (Safe-Lock Tubes 2.0 mL, Eppendorf SE, Hamburg, Germany) filled with 900 µL of sterile sodium chloride solution (NaCl 0.9%, B. Braun SE, Melsungen, Germany). A total of 100 µL of the phage suspension was added to the first tube. After completing the dilution series, 10 µL of each dilution step was placed onto a labelled area of an agar plate, which was covered with a uniform bacterial lawn of the host strain, followed by incubation for 24 h. The lytic zones at each dilution step were counted, and the bacteriophage titre was calculated using the following formula [42]:

$$\mathrm{Bacteriophage}\;\mathrm{titer}\left(\frac{\mathrm{PFU}}{\mathrm{ml}}\right)=\;\frac{\mathrm{Nr}.\;\mathrm{of}\;\mathrm{bacteriophage}\;\mathrm{plaques}\;\mathrm{formed}\;∙\;\mathrm{Dilution}\;\mathrm{factor}}{\mathrm{Volume}\;\mathrm{of}\;\mathrm{bacteriophage}\;\mathrm{sample}\;\left(\mathrm{ml}\right)}$$

Prior to experimental use, bacteriophages were freshly produced, and their titre measured to ensure robust lytic activity.

### Morphological analysis by transmission electron microscopy

The virion morphology was visualised as previously described using transmission electron microscopy (TEM) with the negative staining technique [40]. In brief 15 µL of CUB_EF02 suspension was applied to parafilm, then transferred to a carbon-coated Ni-mesh grid (G2430N; Plano GmbH, Wetzlar, Germany). After 10-15 min of adsorption at room temperature, the grid was washed with distilled water, stained with 1% uranyl acetate (SERVA Electrophoresis GmbH, Heidelberg, Germany) for 20 s, and excess stain removed. The grids were air-dried and imaged by TEM using an electron microscope (Zeiss EM 906, Carl Zeiss Microscopy Deutschland, Oberkochen, Germany) at 80 kV [40]. Using ImageJ (ImageJ, National Institutes of Health (NIH), Bethesda, USA), the head diameter, tail diameter, and tail length of a total of 10 phages were measured and reported as mean ± SD.

### DNA sequencing using the sanger method

The DNA of the isolated phage CUB_EF02 was analysed by Eurofins Genomics Europe Shared Services GmbH (Ebersberg, Germany) using sanger sequencing with the dideoxy method. Different liquids were used to induce the lysis of the phages. This disintegration released the DNA into the solution. Ethanol was added to clean and concentrate the phage DNA, causing the DNA to precipitate [43]. Afterwards, sanger sequencing was performed as previously described 44. For chain termination, dideoxynucleotide triphosphates (ddNTPs) were labelled with a specific colouring agent. Using a 96-Capillary Array DNA sequencer (ABI 3730xl DNA Analyser, SeqGen, Inc., Los Angeles, USA), the DNA was separated according to its molecular weight during gel electrophoresis, and the base sequence was documented through analysis of the colouring agent.

As previously described [45], the Unicycler [46] on the PATRIC v3.6.6 server [47] was used to assemble the raw sequencing data. BLASTn [48] was used to identify the most related bacteriophages. Annotation was performed with RASTtk [49] on the PATRIC server followed by manual annotation using BLASTp and UniProt KB. Easyfig [50] was used to visualise a genome map. All data were submitted to NCBI GenBank under accession number PV296169.

### Biofilm time-killing assay

A time-killing assay was performed to quantify the antimicrobial effect of CUB_EF02, using sterile 4 mm sintered porous glass beads (ROBU, Hattert, Germany). Two independent biological replicates were conducted, with two samples for each time point (0 h, 2 h, 4 h, 6 h, and 24 h) and two additional growth controls. A total of 40 sterile glass beads were placed in sterile 24-well plates (Falcon® 24-well, Corning, NY, USA), one bead in each well, and covered with 990 μL of TSB and 10 μL of an overnight culture of EF02 (ratio 1:100). The glass beads were incubated on a shaker (150 rpm) for 24 h, then dip-washed in 0.9% sterile sodium chloride to remove non-adherent bacteria and transferred to sterile 2 mL tubes. For the growth control, glass beads were covered with sterile PBS and TSB. The other beads were inoculated with 10^8^ PFU/mL of phage suspension and incubated for 2 h, 4 h, 6 h, and 24 h. Samples were taken out of the incubator at specific time intervals, and the biofilm was removed by sonicating the tubes for 10 min at 40 kHz and 0.2 W/cm² (BS 14.2, Bandelin electronic GmbH & Co. KG, Berlin, Germany). Afterwards, the glass beads were extracted using sterile tweezers, and the tubes were centrifuged at 27190 × g for 1 min at 4 °C. Then, the supernatant was discarded, and the bacterial pellet resuspended in sterile PBS. This process was repeated four times to ensure the complete removal of bacteriophages from the bacterial suspension. Next, a six-fold serial dilution of the remaining bacterial suspension was pipetted onto TSA plates and incubated for 24 h. Then the bacterial colonies were counted, and the concentration was calculated using the following formula [40]:

$$\mathrm{Bacterial Count}\left(\frac{\mathrm{CFU}}{\mathrm{mL}}\right)=\frac{\mathrm{No}.\mathrm{of colonies counted}∙\mathrm{dilution factor}}{\mathrm{volume in mL spread on culture plate}}$$

The standard deviation (SD) for data evaluation was calculated and illustrated in graphs as bacterial count (CFU/mL) over time (h) using GraphPad Prism 6 software (GraphPad Software, La Jolla, CA, USA). Significant differences were determined using t-tests. *P*-values < 0.05 were considered significant.

### Preparation of the root canal model *ex vivo*

#### Sample preparation

**Figure 1.**
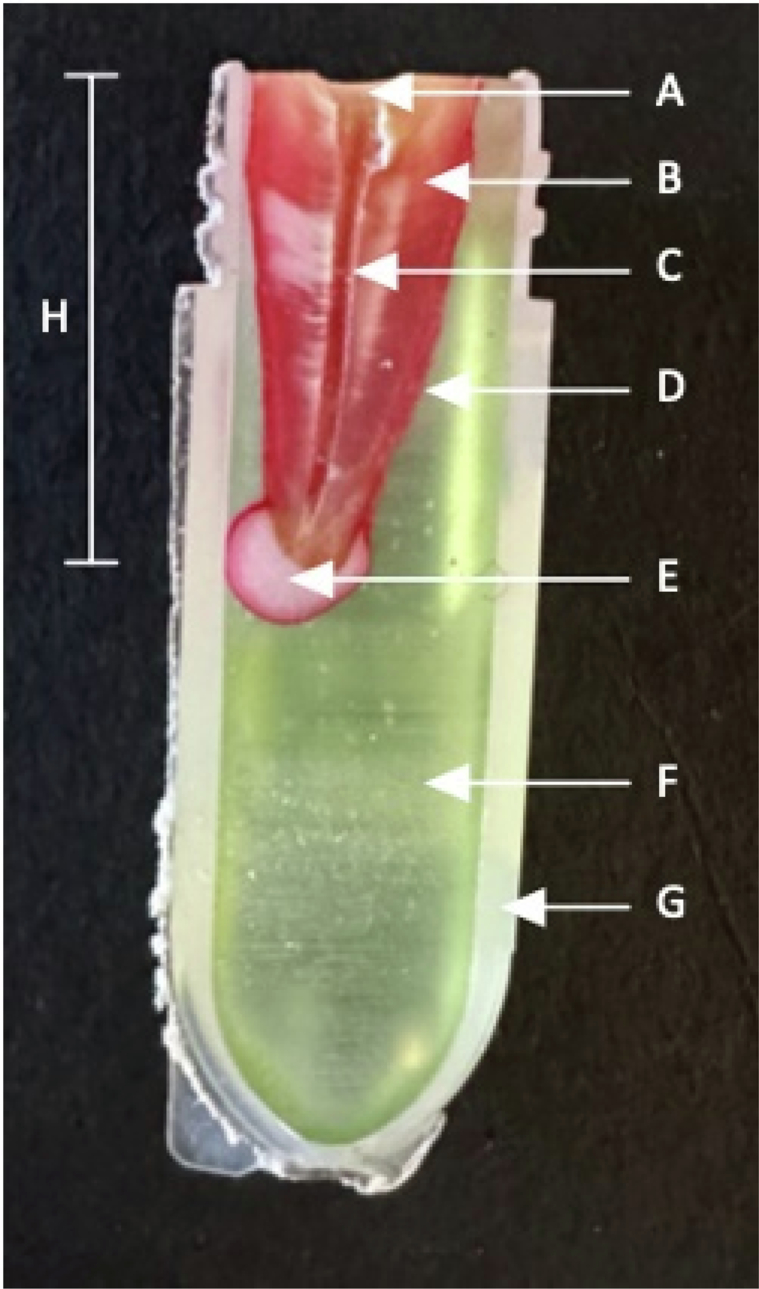
Longitudinal cross-sectional view of the *in vitro* root canal model showing a tooth shortended to 19 mm and embedded in resin; A, coronal reservoir; B, tooth; C, prepared root canal; D, red nail polish; E, glass ionomer cement; F, embedding resin; G, CryoPure tube; H, 19 mm length.

Thirty-six single-rooted human teeth, acquired with written informed consent under an ethics-approved protocol (EA4/102/14) by the Ethical Review Committee of the Charité –Universitätsmedizin Berlin, were collected at Charité—Universitätsmedizin Berlin, Dept. of Operative, Preventative and Paediatric Dentistry, Berlin, Germany. Only teeth with a distance of ≥ 19 mm between the apex and the cementoenamel junction, with closed apices, no sign of cracks or root fractures, or root caries were selected to prepare *in vitro* root canal models (Figure 1). From all teeth, the crowns were removed, and roots were cut to a length of 19 mm using an angle grinder (EXAKT Trennschleifsystem 300 CL, EXAKT Vertriebs GmbH, Nordersedt, Germany). Thereafter, coronal reservoirs (2 × 2 x 1 mm) were prepared with sterile diamonds (2 mm diameter, 801.314.029 VPE 5, Komet Dental Brasseler GmbH & Co. KG, Lemgo, Germany) at the orifice of the root canal. The apexes were sealed with a glass ionomer cement (GIC, Ketac™ Cem Maxixap™, 2 M Deutschland GmbH, Neuss, Germany). Then, the root surface was covered with red nail polish (Express Finish Nagellack, L'Oréal, Düsseldorf, Germany) to prevent any leakage through dentinal tubules or lateral canals. After the nail polish was dried, the roots were embedded in epoxy resin (Technovit 4701, Heraeus Kulzer, Hanau, Germany), covered by a CryoPure tube (1.8 mL, SARSTEDT AG & Co. KG, Nürmbrecht, Germany). The CryoPure was marked with different colours on three sites to indicate locations for the sampling of dentine-adherent bacteria (T0, T1, T2). The root canals were prepared as follows: 1. Coronal flaring with SX ProTaperNext file (ProTaper® Universal SX file, Dentsply Sirona GmbH, Bensheim, Germany) 2. The Apex was located by using C-Files ISO 10 (STERILE C-PILOT Feile ISO 010, VDW Dental GmbH, München, Germany). 3. The root canals were prepared with the ProTaper Next system (X-SMART™, Dentsply Sirona GmbH, Bensheim, Germany) using the files X1, X2, X3, and X4 (ProTaper® Universal X1-4 file, Dentsply Sirona GmbH, Bensheim, Germany) while flushing the canals with 2 ml 0.9% sterile saline for 30 sec after every file preparation. Afterwards, the smear layer was removed by rinsing for 2 min with 10 mL 17% EDTA (CanalPro™ EDTA 17%, COLTENE Holding AG, Altstätten, Switzerland). Finally, the canals were irrigated with 10 mL of 0.9% sterile saline to eliminate EDTA.

#### Tooth sterilisation

Once prepared, the tooth samples were added to 500 mL laboratory containers (Laboratory bottle 21 801 44 59, DURAN GROUP GmbH, Mainz, Germany) filled with brain heart infusion (BHI) (Hirn-Herz-Glucose-Bouillon, Carl Roth GmbH + Co. KG, Karlsruhe, Germany) and subsequently autoclaved (Tuttnauer Autoclave—Steam Steriliser, Tuttnauer Europe B.V., Breda, Netherlands).

One hundred µL of the culture suspension from each tooth was added to a TSA plate and incubated for 24 h to check for contaminants.

#### Dentine-adherent biofilm establishment

Ten μl of the EF02 ONC was pipetted into each root canal model and supplemented with TSB until the liquid reservoir was filled (approximately 40 μl). The CryoPure tube was closed airtight and incubated overnight. After 24 h, the CryoPure tubes were reopened to refill the root canal with TSB until the liquid reservoir was filled again. The samples were then incubated for a further 24 h. A biofilm formation assay was performed to determine the time required for a homogeneous dentine-adherent biofilm to develop in the root canal. Therefore, the dentine-adherent biofilms of 13 models were analysed after 1 h, 2 h, 3 h, 4 h, 5 h, 6 h, 12 h, 1 d, 2 d, 3 d, 5 d, 7 d, and 10 d. After incubation, the canals were rinsed with 0.9% NaCl to remove planktonic bacteria. Thereafter, a sterile Hedstroem file ISO 25 (STERILE Hedstroem Files ISO 25, VDW Dental, München, Germany) was stroked once from apical to coronal to sample dentine-adherent bacteria. The file was placed in a 2 mL tube containing 2 mL sterile PBS buffer and the biofilm was removed by sonication for 10 min at 40 kHz and 0.2 W/cm². Afterwards, a six-fold serial dilution was performed and spotted onto a TSA plate. The plates were incubated for 24 h, bacterial colonies were counted, and CFU/mL were calculated. The standard deviation (SD) for data evaluation was calculated and illustrated in graphs as bacterial count (CFU/mL) over time (h) using GraphPad Prism 6 software.

### Fluorescence *in situ* hybridisation (FISH) of the bacterial biofilm

Four teeth were subjected to FISH to visualise the intracanal biofilm architecture. Specimens were fixed in paraformaldehyde (4%), embedded in cold polymerising resin (Technovit 8100; Heraeus Kulzer, Hanau, Germany) and cut into 1 mm thick sections on a microtome (KDG-95; MeProTech, Leiden, The Netherlands). The sections were then re-imbedded in resin (Technovit 8100) and further cut to a slice thickness of 7 µm using a hard tissue microtome (Polycut Legacy; Gigatome, Roggenburg, Germany). The thin slices were spread out on object slides (Polysine; Menzel-Gläser, Braunschweig, Germany), dried and hybridised following a protocol modified from Del Rey *et al.* [51]. Formamide-fixed cultures of *E. faecalis* (DSM 2570) and the typical endodontic pathogens *Actinomyces naeslundii and Fusobacterium nucleatum* were used as positive and negative controls, respectively.

Probes ENF191 (5’- GAA AGC GCC TTT CAC TCT TAT GC-3’), 5’ end-labelled with Cy5 and specific for *E. faecalis*, and EUB338 (5’-GCT GCC TCC CGT AGG AGT-3’), end-labelled with Atto555 and targeting most bacteria were employed, both at a working concentration of 100 ng/µL. Specimens were treated with lysozyme (9 min at 37 °C; 70 U/mL in 100 mM Tris/HCl, pH 7.5, and 5 mM EDTA; Merck, Søborg, Denmark) to permeabilize bacterial cells, and then dehydrated by serial ethanol washes (50%, 75%, 100%) for three min each. Hybridisation was performed for 100 min at 46 °C and 20% formamide, followed by a 15-min stringency wash at 48 °C and a 3-sec rinse in ice-cold Milli-Q water.

### Confocal microscopy analysis of the bacterial biofilm

Biofilm and control samples were imaged with a confocal microscope (Zeiss LSM 700). ENF191-Cy5 was excited using a 639 nm laser line and detected from 644-800 nm, while EUB388-ATTO550 was excited at 555 nm and detected from 560-600 nm. Autofluorescence from dentin was excited with a 488 nm laser line and detected from 300-550 nm. To avoid spectral bleed-through, ENF191-Cy5/autofluorescence and EUB338-ATTO550 were imaged in separate subsequently recorded channels. Images were acquired with a 63x (NA = 1.4) objective (Plan Apochromat, Zeiss), with an optical slice thickness of 1.0 µm, a frame size of 1192 × 1192 pixels and a pixel dwell times of 1.09 µs, respectively. Images were acquired in arbitrarily selected locations, for qualitative analysis of the biofilm structure only. Linear averaging (*n* = 4) was applied for noise reduction.

### Reduction of the dentine-adherent bacteria in the *in vitro* root canal model

**Figure 2.**
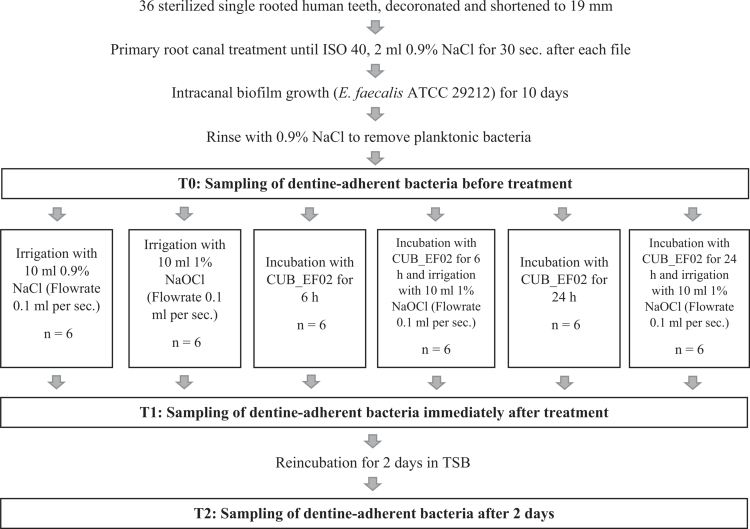
Flow chart of the study design, detailing treatment steps and sampling time points. *n*, number; NaCl, sodium chloride; NaOCl, sodium hypochlorite; T, time point; TSB, tryptic soy broth.

After 10 days of *E. faecalis* biofilm growth, 36 samples were randomly assigned to six different experimental groups (*n* = 6 per group). Each group consists of six independent root canal specimens, which were treated and processed separately. A schematic representation of the groups and the different treatments is shown in Figure 2. Group 1 received irrigation with 10 mL 0.9% NaCl (flow rate: 0.1 mL/s, negative control). Group 2 was irrigated with 10 mL 1% sodium NaOCl (positive control) at the same flow rate. Group 3 underwent intracanal incubation with CUB_EF02 for 6 h without subsequent irrigation. Group 4 received intracanal incubation with CUB_EF02 for 6 h followed by irrigation with 10 mL 1% NaOCl (0.1 mL/s). Group 5 was incubated with CUB_EF02 for 24 h without irrigation, whereas Group 6 was incubated with CUB_EF02 for 24 h followed by irrigation with 10 mL 1% NaOCl (0.1 mL/s)Before the treatments were performed, the samples were rinsed with 10 mL sterile 0.9% saline to remove planktonic bacteria. Following incubation with bacteriophages and irrigation with NaOCl, the canals were rinsed with 5 mL of NaCl for 30 seconds to eliminate residual antimicrobial agents. Dentine samples were taken with sterile Hedstroem files (ISO 25) at three different time points: before treatment (T0), right after treatment (T1), and after two days of regrowth (T2). Files were stroked once from apical to coronal along the root canal wall in the locations marked on the cryotube and then placed in a tube filled with 2 mL sterile PBS buffer. To remove the bacteria from the file, the tubes were sonicated for 10 min at 40 kHz and 0.2 W/cm². Afterwards, a six-fold serial dilution was performed, plated on TSA and incubated for 24 h. CFU counts were determined as described above.

### Statistical analysis

For each treatment group, the logarithmic reduction factor (LRF) was determined by calculating the difference between bacterial counts in CFU/mL at the time points T0 and T1 (LRF_red._), T2 and T1 (LRF_reg._), and T0 and T2 (LRF_total_).

A one-way ANOVA was carried out to determine the effect of the different irrigation solutions on LRF_red._, LRF_reg._, and LRF_total_ using SPSS Statistics 25 (IBM Deutschland GmbH, Böblingen, Germany). A post-hoc test (Tukey´s HSD) was performed to investigate differences between the groups' log-reduction. The significance level (*α*) was set at *p* < 0.05.

### Evaluation of bacterial resistance

After treatment and sampling (T1), fresh TSB was added and teeth with biofilms were incubated again for 48 h. Dentine-adherent bacteria were sampled and CFU counts determined, as described previously (T2). Single bacterial colonies (colony-variants) were recovered to evaluate the susceptibility of the treated EF02 and the untreated host strain (parental strain) to phage CUB_EF02. Using the bacterial suspension extracted from T2, an ONC was produced for each sample from groups 3, 4, 5, and 6 and incubated for one day. Using MTA-TSB, a specific bacterial lawn was created on a TSA plate. Then 10 µL of the CUB_EF02 suspension used during treatment was poured onto the bacterial lawn, incubated for 24 h and checked for clear lytic zones to assess bacterial resistance.

## Results

### Plaque morphology and TEM visualisation

**Figure 3.**
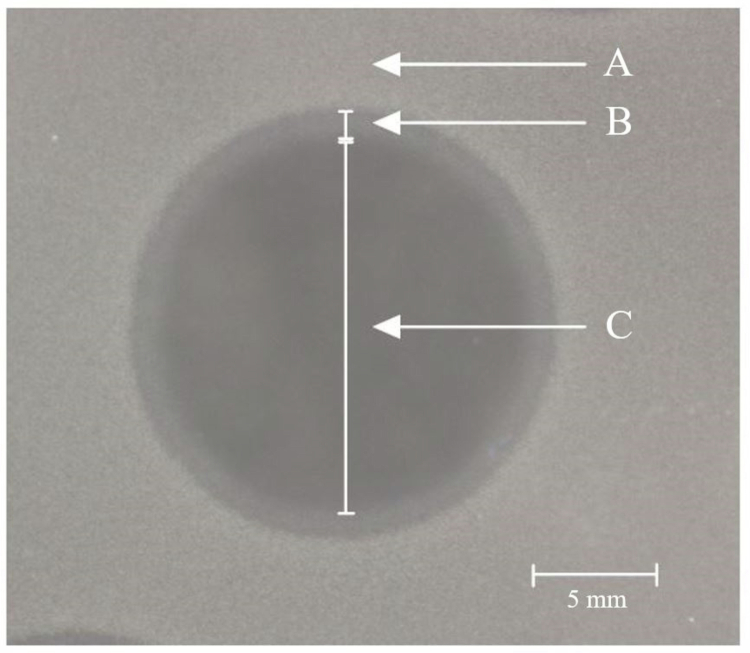
Plaque morphology of the phage CUB_EF02 on an agar plate covered with a homogenous bacterial lawn of *Enterococcus faecalis* EF02 (A); B, halo zone; C, lytic plaque; Scale bar = 5 mm.

The phage CUB_EF02 had a strong lytic activity against EF02, as confirmed by a large and transparent plaque morphology when dropped onto an agar plate covered with a homogeneous bacterial lawn of EF02. Around the lytic circle, an opaque halo zone was observed, which exhibited increased transparency compared to the surrounding homogeneous bacterial lawn (Figure 3).

**Figure 4.**
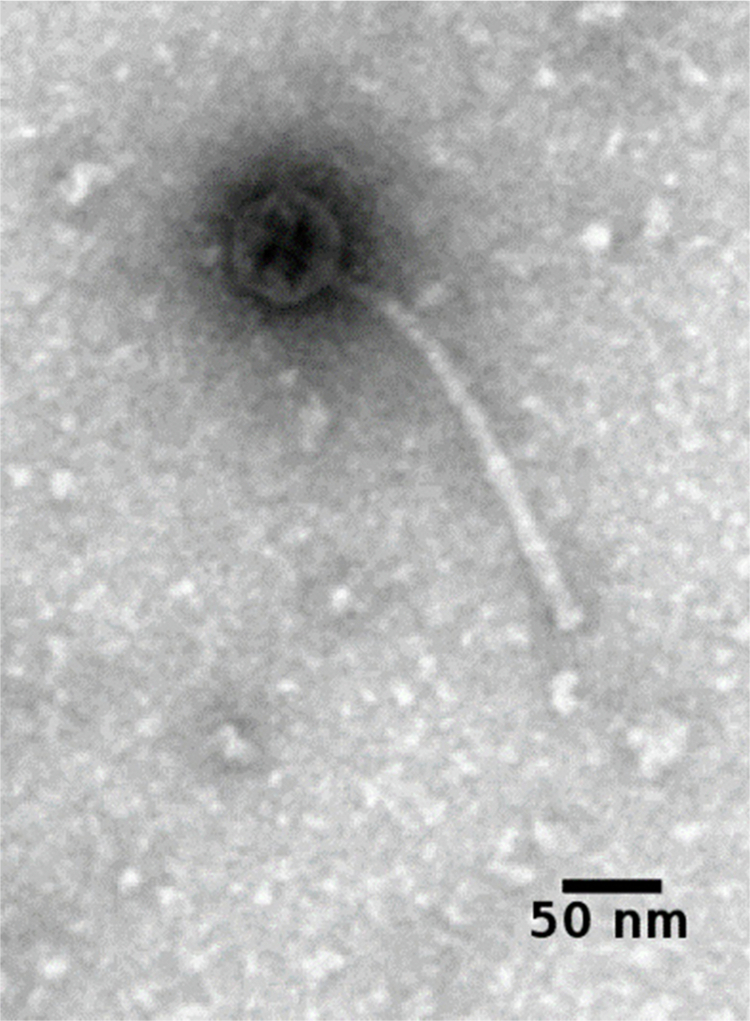
A transmission electron micrograph shows image of a bacteriophage CUB_EF02 virion with a hexagonial head and a long flexible tai. Scale bar = 50 nm.

Transmission electron microscopy showed that CUB_EF02 is a siphovirus and belongs to the group B morphotype in Bradley's classification [52] (Figure 4). It has a hexagonal head with a relatively flexible, long tail, without a contractile apparatus [52]. Measurements of CUB_EF02 revealed a head diameter of approximately 52,5 ± 5,02 nm. The tail exhibited a mean width of 8.7 ± 1.64 nm and an overall length of 197.8 ± 19.95 nm, measuring approximately four times the head diameter.

### DNA sequencing using the sanger method

The genome of CUB_EF02 was determined using Sanger sequencing and showed a dsDNA with 38,366 bp. The sequence was compared to the entries in the BLASTn database and showed 95.1% identity with *Enterococcus* phage vB_EFaS_TV217 (MT627482.1; 98% query coverage) [53] and 95.0% identity with *Enterococcus* phage phiSHEF10 (OL799256.1; 97% query coverage).

**Figure 5.**
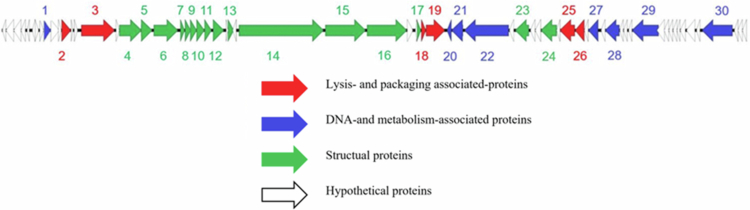
Linear CUB_EF02 genome map with 30 numbered arrows representing coding sequences and a legend for protein types red arrows: genes coding for lysis-associated and packaging proteins; blue arrows: DNA- and metabolism-associated proteins a; green arrows: structural proteins; white arrows: hypothetical proteins; the arrows are numbered and correspond to proteins listed in Table 1.

Structural annotation of the genome identified 67 coding sequences (Figure 5, Table 1), and no lysogen-associated proteins were found. This result was also verified using the Phage AI logarithm, which also predicted a lytic life cycle [54]. In addition, no known virulence proteins were encoded on the phage genome (verified with VirulenceFinder [55]. This and the fact that the genome predicted a lytic life cycle make the phage CUB_EF02 potentially suitable for phage therapy.

**Table 1.** This table summarises the proteins associated with the numbered arrows indicated in the genome map (Figure 5).

Number	Protein	Number	Protein
1	Phage-associated homing endonuclease	16	Phage tail assembly protein
2	Phage terminase small subunit	17	Phage tail fibre
3	Phage terminase large subunit	18	Phage holin
4	Phage portal connector protein	19	Phage lysin, *N*-acetylmuramoyl-L-alanine amidase
5	Phage maturation protease	20	Glutaredoxin, phage associated
6	Phage major capsid protein	21	DNA modification
7	Phage tail tube protein	22	DNA polymerase B region
8	Phage DNA packaging protein	23	Neck protein
9	Head tail adaptor protein	24	DUF1351 domain-containing protein
10	Neck protein	25	MBL fold metallo-hydrolase
11	Head-tail joining protein	26	Putative HNH homing endonuclease-like protein
12	Phage major tail protein	27	Nucleotide kinase
13	Distal tail protein	28	DNA polymerase/primase
14	Phage tail tape measure	29	DNA helicase
15	Distal tail protein	30	DNA primase

### Time-killing assay of EF02 biofilms on glass beads

**Figure 6.**
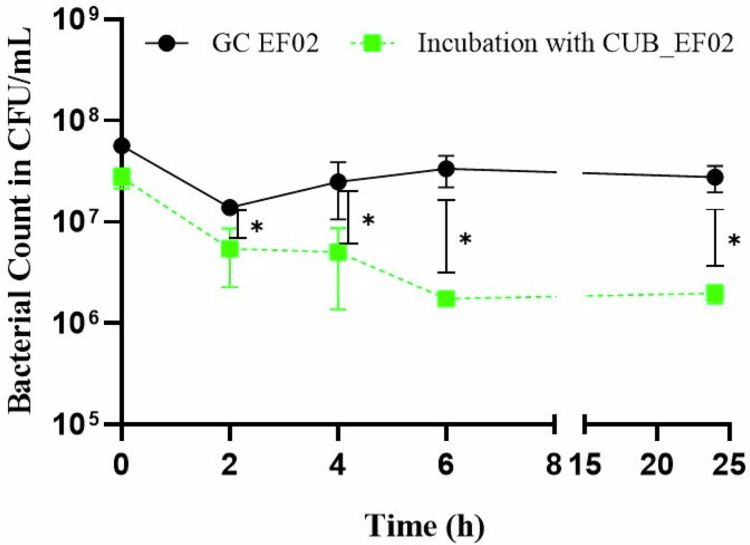
Time-killing curve of untreated (GC) and phage-treated (10^8^ PFU/mL) biofilms from host strain *Enterococcus faecalis* EF02 monitored at five different time points (0 h, 2 h, 4 h, 6 h, and 24 h). Data are expressed as mean ± SD. *P*-values < 0.05 were considered significant (*) using t-tests; CFU, colony forming units; T, time.

The time-killing kinetics of EF02 biofilms grown on porous glass beads showed significant reductions after exposure times of 2 h, 4 h, 6 h, and 24 h with CUB_EF02 (Figure 6). The effect increased to 6 h of exposure (1.35 ± 0.2 CFU log reductions) and then reached a plateau. Although the reduction after 24 h was lower, it was still similar to that after 6 h. The 1.35 ± 0.2 -log CFU reduction indicated that fewer than 10% of the bacteria remained on the surface of the glass beads.

### *In vitro* biofilm establishment in root canals

**Figure 7.**
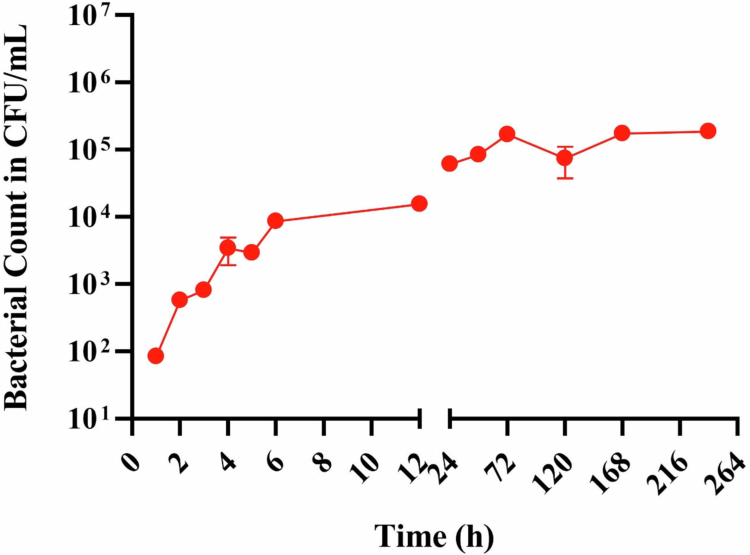
FA linear graph shows the formation of the dentine-adherent *Enterococcus faecalis* EF02 biofilm in the *in vitro* root canal model, measured as CFU/mL over time in hours; CFU, colony forming units; T, time; error bars = SD.

After 1 h of incubation, some bacteria were already detectable (83.3 CFU/mL) on the root canal wall, while the bacterial count increased steadily to 10^5^ CFU/mL after an incubation period of 3 days. With minor fluctuations, the number of CFU in the dentine-adherent biofilms remained constant at 10^5^ CFU/mL until 10 days of incubation (Figure 7).

### Fluorescence microscopy of the dentine-adherent bacterial biofilm

**Figure 8.**
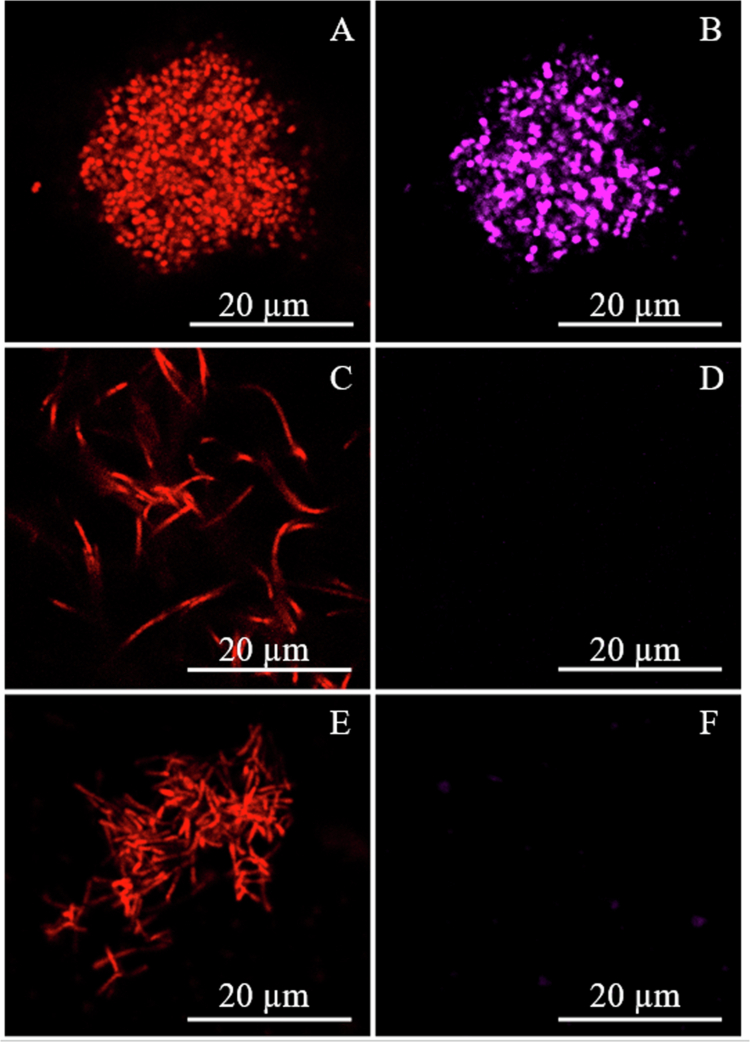
Fluorescence *in situ* hybridisation of *Enterococcus faecalis* and the control strains *Fusobacterium nucleatum* and *Actinomyces næslundii. E. faecalis* was targeted by both the universal probe EUB338 (A) and the species-specific probe ENF191 (B). *F. nucleatum* and *A. naeslundii* were only visualised by EUB 338 (C, E), but not by ENF191 (D, F). Scale bars = 20 µm.

**Figure 9.**
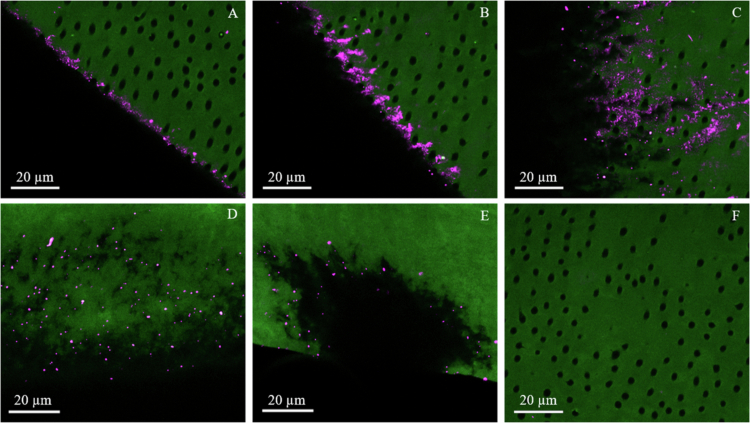
Fluorescence *in situ* hybridisation of root canals infected with *Enterococcus faecalis. E. faecalis* (magenta) colonised all parts of the root canal system. In some areas, the biofilms formed a compact layer on the root canal wall (green) with a thickness of 5-10 µm (A and B). In other areas, the organisms invaded the root dentin up to a depth of 80 µm (C-E). Deeper layers of the root dentin were unaffected by *E. faecalis* (F). Scale bars = 20 µm.

FISH demonstrated the successful biofilm formation of *E. faecalis* in the root canals. Probe ENF191 targeted *E. faecalis*, but not the negative controls *F. nucleatum* or *A. naeslundii* (Figure 8). All parts of the examined root canals were colonised by *E. faecalis.* In some areas, biofilms were attached as a compact layer with a thickness of 5-10 µm (Figure 9A, B). In other areas, especially when the canal wall was jagged due to mechanical treatment, *E. faecalis* invaded the tissue and penetrated up to 80 µm into the dentin (Figure 9C​​​​​, D, E). Deeper layers of the root dentin were not affected by *E. faecalis* (Figure 9F).

### Reduction of the dentine-adherent bacteria in the root canal model

After ten days of biofilm growth treatments were performed and dentine-adherent bacteria quantified by CFU counts, straight after treatment and after two days of re-growth.

Rinsing with NaCl did not result in a reduction of the dentine-adherent biofilm at T1 (LRF_red._), and consequently, no significant increase was observed after re-growth at T2 (LRF_reg._). CUB_EF02 treatment for 6 h and 24 h significantly reduced the number of recovered cells compared to control (group CUB_EF02 (6 h) *p* = 0.006 and group CUB_EF02 (24 h) *p* = 0.021, Tukey HSD), with no significant difference between exposure times (*p* = 0.994, Tukey HSD). Treatment with NaOCl resulted in a reduction of almost 3-log (99.9%), which was significantly greater than after treatment with CUB_EF02 for 6 h or 24 h (group CUB_EF02 (6 h) *p* = 0.021 and group CUB_EF02 (24 h) *p* = 0.005, Tukey HSD). Combined treatment with NaOCl and CUB_EF02 for either 6 h or 24 h slightly increased the effect of NaOCl without reaching statistical significance (group CUB_EF02 (6 h) + NaOCl *p* = 0.0168 and group CUB_EF02 (24 h) + NaOCl *p* = 0.719, Tukey HSD) (Table 2).

**Table 2.** Logarithmic reduction factors for dentine-adherent bacteria in the different treatment groups; LRF, logarithmic reduction factor; NaCl, sodium chloride; NaOCl, sodium hypochlorite; CUB_EF02, phage against *Enterococcus faecalis*; T0, baseline; T1, immediately after treatment; T2, after 48 h of re-incubation; Superscript letters illustrate statistically significant differences between groups using the compact letter display (CLD) method (one-way ANOVA, Tukey HSD).

Irrigation solution	NaCl	NaOCl	CUB_EF02 6 h	CUB_EF02 6 h + NaOCl	CUB_EF02 24 h	CUB_EF02 24 h + NaOCl
Dentine-adherent LRF_red._ (T0-T1) (Mean ± SD)	−0.21 ± 0.89^a^	2.98 ± 0.77^c^	1.50 ± 0.23^b^	4.05 ± 1.17^c^	1.27 ± 0.32^b^	3.59 ± 0.71^c^
Dentine-adherent LRF_reg._ (T2-T1) (Mean ± SD)	−0.50 ± 0.75^a^	1.87 ± 0.75^bd^	0.37 ± 0.46^ac^	2.38 ± 0.46^b^	0.74 ± 0.48^acd^	1.65 ± 0.54^bc^
Dentine-adherent LRF_total._ (T0-T2) (Mean ± SD)	0.29 ± 1.14^a^	1.11 ± 0.52^acd^	1.14 ± 0.32^ad^	1.58 ± 0.55^bcd^	0.51 ± 0.59^ac^	1.95 ± 0.28^bd^

The LRF_reg._ expresses the number of bacteria that re-grow after treatment (T2-T1), while the LRF_total_ represents the reduction after re-growth compared to the initial pre-treatment counts (T0-T2). Teeth that were only treated with CUB_EF02 showed significantly lower bacterial re-growth than teeth treated with NaOCl (*p* = 0.047, Tukey HSD). Absolute bacterial counts at T2, however, were similar for NaOCl-treated teeth (with and without additional phage treatment) and teeth that were solely treated with phages (*p* = 1.00, Tukey HSD). The lowest cell counts after re-growth were observed for treatment with CUB_EF02 and NaOCl, but the differences were not significant compared to teeth treated with NaOCl only (*p* = 0.838, Tukey HSD) (Table 2).

### Evaluation of bacterial resistance

**Figure 10.**
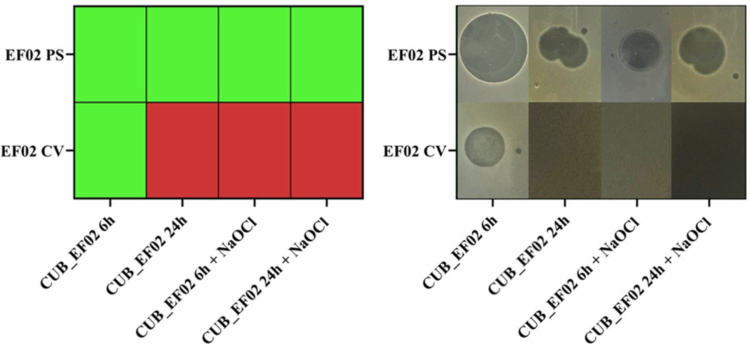
Heatmap (left) and representative images (right) showing the results from the resistance testing of colony variants after phage treatment compared to the parental host strain; in green, lytic zones present; in red, lytic zones absent; colony variant (CV), extracted bacteria from T2; PS, parental strain; NaCl, sodium chloride; NaOCl, sodium hypochlorite; CUB_EF02, phage against *Enterococcus faecalis*.

A top agar overlay spot assay was performed to ascertain resistance of the colony variant bacteria to CUB_EF02. Clear lytic zones were observed on all agar plates of the parental strain, indicating continued susceptibility, as well as for colonies treated with CUB_EF02. In contrast, the colony variants treated with CUB_EF02 for 24 h and those co-treated with CUB_EF02 and NaOCl did not show any lytic zones on the agar plates, which indicates a possible development of resistance (Figure 10).

### Discussion

A lytic bacteriophage, CUB_EF02, targeting *E. faecalis* ATCC 29212, was isolated and presents a siphovirus morphology in the TEM analysis. Its genome consists of 67 coding sequences and reveals two hits in the BLASTn database. In a time-killing assay, the optimal incubation time for CUB_EF02 against EF02 was determined to be 6 h, resulting in a significant bacterial reduction of 1.35 ± 0.2 log.

The null hypothesis of the study was rejected. The antimicrobial effect, represented as LRF_red._, varied significantly depending on the rinsing solution used. Immediately after treatment (T1), the groups incubated with CUB_EF02 have shown a significant reduction of the biofilm, regardless of whether the incubation was performed for 6 h or 24 h. Nevertheless, the groups rinsed with NaOCl were significantly more effective against the biofilm compared to the samples incubated with bacteriophages. On the one hand, the results demonstrate the strong antimicrobial effect of NaOCl and emphasise its essential role in state-of-the-art root canal treatment. On the other hand, the results also showed that incubation with bacteriophages alone (1.50 log reduction) was not sufficient to eliminate the biofilm during root canal treatment, as this would leave more than 5% of the bacteria in the root canal system viable.

The antimicrobial effect of NaOCl is directly correlated with the volume used [56]. Ten mL of a 1% NaOCl solution contains significantly more antimicrobial molecules than 40 μl CUB_EF02 suspension contains antimicrobial phages. This difference in volume, and consequently in the number of active particles, may account for the significantly stronger antimicrobial effect of NaOCl compared to CUB_EF02. The volume of phage suspension used cannot be arbitrarily increased, as it is limited by the size of the root canal. Furthermore, due to the phage production methodology employed, it was not possible to achieve a phage concentration higher than the 10^8^ PFU/mL that was utilised. In this aspect, it is important to note that the antimicrobial effect of bacteriophages strongly depends on the titre of the applied solution. Higher titres increase the multiplicity of infection (MOI) and thereby the probability of successful host cell infection and subsequent phage replication and progeny production [57]. As shown in previous studies, advanced concentration techniques such as PEG precipitation followed by dialysis or filtration [58] or anion exchange chromatography [59] enable the production of phage suspensions with titres of up to 10¹¹ - 10¹² PFU/mL. The low phage concentration used in the present study may therefore have limited the observed therapeutic outcome, especially in the monophage therapy groups.

The present study used a rather low concentration of NaOCl (1%). In clinical practice, higher concentrations (3–5.25%) with increased antimicrobial and virucidal activity are more commonly applied [60], but we opted for a lower concentration to be able to measure additive effects of NaOCl and phage therapy. When both therapeutic approaches are combined, it is important to strictly separate the application of bacteriophages and NaOCl to prevent inactivation of the phages by NaOCl. Moreover, phages should be applied prior to NaOCl, as they weake the biofilm structure and thereby facilitate deeper penetration of NaOCl into the biofilm.

This result contrasts with the findings of other studies. For example, Khalifa et al. 2016, using an *ex vivo* two-chamber bacterial leakage model of human teeth, demonstrated a reduction of the *E. faecalis* biofilm by up to 8 logs upon phage treatment [21], which represents a much more promising outcome for phage application in endodontics. The differences between both studies may, in part, be explained by to the use of different root canal models, which substantially limits the comparability of results. While Khalifa et al. 2016 determined the bacterial concentration, and thus the log reduction, by assessing the turbidity of the culture medium surrounding the model, this study detected the dentine-adherent biofilm and disregarded planktonic bacteria. Additionally, a different bacteriophage was used, which may exhibit distinct antimicrobial efficacy and incubation times compared to the one used in our study. Nevertheless, this study demonstrates an additive antimicrobial effect of the combined treatment with phages and NaOCl. Samples treated with these suspensions showed a reduction of slightly more than 4 logs and eliminated 10 times more bacteria than NaOCl alone. Bacteria gain a certain degree of protection from surrounding substances by forming and aggregating into a biofilm. One of the key components of this protection is the extracellular polymeric substance (EPS) matrix, which consists of extracellular polysaccharides, proteins, nucleic acids, and other substances that prevent the penetration of NaOCl into the deeper layers of the biofilm, allowing only the peripheral layers to be eliminated [61]. After the removal of the antimicrobial agent, the biofilm can re-form from the intact lower layers. Bacteriophages infect bacteria in the upper biofilm layers, replicate, and release their progeny into the environment, enabling them to penetrate deeper layers and infect bacteria residing there.

Furthermore, genome sequencing revealed the presence of base sequences encoding holin proteins and phage lysins on the dsDNA of CUB_EF02. These proteins are produced during the final stage of the lytic cycle of bacteriophages within the host cell [60]. The holin proteins create pores in the inner membrane of the bacterial cell wall, allowing the endolysins, which are peptidoglycan-degrading enzymes, to access the peptidoglycan layer [62]. There, they catalyse its degradation, leading to the lysis of the host cell [63]. Consequently, the endolysins and their progeny are released into the deeper layers of the biofilm, where they continue to exert their antimicrobial effects. Additionally, several studies [41,64] have demonstrated that phages carry specific enzymes, such as EPS depolymerases and endo-glycanohydrolases, on the surface of their tail, which degrade extracellular polysaccharides, making the bacteria more accessible and destabilising the biofilm. Nevertheless, there is no clear evidence that DNA sequences coding for such proteins are present in the isolated CUB_EF02 genome when compared to existing databases. The genome map does not contain identifiable gene sequences that are classified as hypothetical proteins, which could potentially encode such enzymes. It should be noted that certain proteins may perform enzymatic functions in addition to their structural roles, suggesting that hypothetical proteins identified in the CUB_EF02 genome may require further investigation in this context. Moreover, it has to be considered that there is a clear possibility that not the entire genome of CUB_EF02 has been analysed. However, we have demonstrated that CUB_EF02, when applied to a homogeneous bacterial lawn of the host bacterium, produces halo zones around the lytic plaque. As shown previously [41], the presence of halo zones is a clear indicator of phage-associated EPS depolymerases. Although the halo zones contain as many bacteria as the surrounding uninfected areas, they appear more translucent due to the degradation of the EPS matrix, which weakens the biofilm and facilitates the penetration of antibacterial substances such as NaOCl. These three mechanisms significantly disrupt the biofilm structure and remove its protective effect on the bacteria, allowing NaOCl to penetrate deeper into the biofilm. This may explain why the incubation with bacteriophages followed by rinsing with NaOCl resulted in the highest antimicrobial effect in the study.

The bacterial regrowth observed in all samples of all groups after 2 days of additional incubation clearly demonstrates that it is nearly impossible to achieve complete eradication of all microorganisms within root canals, even when using a simplified laboratory-based biofilm model. The LRF_red._ and LRF_total_ values showed only few isolated significant differences regarding the irrigant used. Therefore, the bacterial recontamination and the irrigation protocol employed can’t be linked. This is particularly supported by the fact that the microbial load present in the root canal at time point T2 differed only minimally among the groups.

The top agar overlay spot assay demonstrated bacterial resistance to CUB_EF02 when incubated for 24 h or when NaOCl and CUB_EF02 were used in combination. Bacteriophages and bacteria have coexisted for millions of years, which is why bacteria can develop resistance to phages in various ways, similar to antibiotics [65]. One of the most common mechanisms of bacterial resistance against phages is the alteration or loss of the bacterial surface protein to which phages bind, or the presence of certain membrane proteins that prevent the insertion of phage DNA [66,67]. Importantly, the present findings also indicate that bacterial resistance developed rapidly, already within 6 hours of incubation. This observation highlights an inherent limitation of monophage therapy, as single-phage applications are particularly susceptible to the emergence of phage-resistant bacterial subpopulations. Consequently, the regrowth observed in all experimental groups is likely, at least in part, associated with this rapid development of resistance, which may have limited sustained antimicrobial efficacy over time. As recent research by Moryl et al. 2023 [68] shows, treatment of biofilms with bacteriophages leads to an increased amount of eDNA in the remaining biofilm. It is known that eDNA present in the biofilm matrix promotes the aggregation of surviving or remaining bacteria and enhances bacterial cell adhesion. Thus, the increase in eDNA in phage-treated biofilms may contribute to renewed bacterial adhesion and proliferation, which could explain why regrowth was observed in all groups at time point T2.

The use of phage cocktails, which consist of different phages that are all lytic against the same host strain, can reduce the potential development of resistance, as it is highly unlikely that bacteria will simultaneously develop resistance to all the phages used [69]. Combining different phages can significantly enhance the antimicrobial effect. However, studies indicate that rare instances of cross-resistance can cause bacteria to become resistant to all phages in a cocktail simultaneously [70]. To explore this issue further, a deeper understanding and investigation of the basic principles of resistance mechanisms are required. In addition to the potential use of phage cocktails to prevent bacterial regrowth, the findings of Shukla et al 2025 [37] should also be considered. As the study shows, two subsequent applications of the phage suspension were sufficient to completely eradicate the bacterial biofilm. Repeated application of the same phage solution therefore represents a promising strategy to counteract bacterial regrowth. Accordingly, the strong regrowth observed in our study may be explained by the single-dose phage application used in our model, which may not have been sufficient to prevent the emergence of resistant subpopulations.

Another approach could be treatment with combinations of phages and antibiotics, which has demonstrated synergistic effects in some studies and has also inhibited biofilm formation [65,71]. However, the mechanism of action of this combination therapy must be thoroughly understood, as some antibiotics, particularly those that disrupt bacterial cell walls or those who affect ribosome can interfere with phage replication and may reduce the effectiveness of the phages by eliminating the host cells in which they can replicate [72]. This should also be addressed in further investigations. Furthermore, the question arises as to what extent the presence of resistance in the remaining bacteria within the canal system to the phage is of clinical relevance, provided there is a sufficiently strong reduction of the biofilm and the subsequent obturation of the canal system and coronal restoration are performed according to proper standards.

Another advantage of bacteriophages is their high specificity towards their host, which means that they are considered non-pathogenic substances and are therefore highly biocompatible. This allows for the potential application of bacteriophage suspensions beyond the apical foramen into the periapical tissue to target and eliminate the bacteria present there. In contrast, due to the strong tissue-damaging effects of NaOCl and the risk of irrigation accidents, it should never be applied beyond the apex. Phages offer a promising treatment option in this regard, especially when considering that *E. faecalis* is present in about half of non-healing periapical lesions.

The clinical application of personalised phage therapy in root canal treatments requires more complex logistics than standardised procedures, as it is necessary to identify the bacteria present in the root canal beforehand to determine which phages to use. Considering the labour-intensive and time-consuming process of isolating bacteriophages from wastewater samples and bearing in mind that the biofilm responsible for apical periodontitis contains a variety of bacteria, the production of phage cocktails to address a complex biofilm seems almost unfeasible. However, with the current state of the art, the first opportunities are emerging to produce bacteriophages *in vitro* through synthetic means tailored to the specific host strain [73]. Although such a device is not yet commercially available, its development has the potential to revolutionise the therapeutic application of bacteriophages in medicine. This device would analyse the DNA of the host bacterium and leverage artificial intelligence to search relevant databases for a pre-sequenced, suitable bacteriophage [74], which would then be synthesised [75] and produced directly in a cell-free system [76]. This underscores the substantial potential of bacteriophages, which, with further research, could profoundly transform the management of bacterial infections, not only within dentistry but across various medical disciplines, offering a promising adjunct to conventional antibiotic therapy.

This study has several limitations. *E. faecalis* is frequently used in experimental endodontic studies due to its ability to survive long-term starvation, its rapid and easy cultivation, and its straightforward biofilm formation [77]. This is further demonstrated by the successful establishment of a biofilm in the *in vitro* root canal model after 3 days of incubation. Notably, an *E. faecalis* monospecies biofilm was established, which does not replicate the complexity and virulence of the polymicrobial biofilm typically associated with secondary apical periodontitis. Our aim was to transfer the antimicrobial effect of the phages, demonstrated through the time-killing assay, to a simplified *in vitro* root canal model. The results indicate that bacteriophages possess antimicrobial activity against mono-species biofilms in the root canal, and further research is needed to explore their efficacy against multi-species biofilms using phage cocktails. Furthermore, we have only assessed the antimicrobial effect of a single phage against laboratory strain. Given the vast diversity of bacteriophages, no general conclusions can be drawn regarding their overall antimicrobial potential. Additionally, it is not possible to predict how the bacteriophage would behave against a clinical *E. faecalis* strain.

Sampling of the dentine-adherent biofilm was performed using Hedstroem files, which were stroked once with constant pressure from apical to coronal. The maximum removal of bacteria from the root canal wall is limited by the size of the file. Moreover, bacteria that have penetrated into the dentinal tubules cannot be eliminated mechanically, meaning they are not accounted for in the calculation of the antimicrobial efficacy of the respective irrigants. Consequently, the study cannot draw any conclusions regarding the effect on these intratubular bacteria. As with the preparation of the root canal models and the mechanical preparation of the root canals, this procedure was carried out by the same operator to ensure standardised experimental conditions, although it represents a potential source of error. Bacterial counts of the samples taken at different time points were determined using CFU, a clear and universally accepted outcome measure [78]. While this method is time-consuming and labour-intensive, it was the most feasible option for our study compared to other available outcome measurement techniques.

## Conclusions

The results of this study show that incubation with bacteriophages resulted in a significant reduction of an *E. faecalis* monospecies dentin-adherent biofilm. However, the antimicrobial effect of bacteriophages was significantly weaker compared to NaOCl. Additionally, the incubation of bacteriophages can lead to bacterial resistance. Whether bacteriophage treatment can enhance the disinfection of infected root canal systems *in vivo* remains to be clarified in future studies.

## Data Availability

The data that support the findings of this study are available from the corresponding author (KS) upon reasonable request.
